# Delayed-Onset Seizure in a Mild Quetiapine Overdose: Report of a Case and Review of the Literature

**DOI:** 10.1155/2018/7623051

**Published:** 2018-05-15

**Authors:** Jason A. Chen, Katherine M. Unverferth, Erick H. Cheung

**Affiliations:** Department of Psychiatry and Biobehavioral Sciences, UCLA David Geffen School of Medicine, Los Angeles, CA 90095, USA

## Abstract

Among atypical antipsychotics, quetiapine is commonly prescribed and considered to have a favorable side effect and safety profile. Here, we report the case of a patient who developed a generalized tonic-clonic seizure 28 hours following ingestion of 1,400 mg of quetiapine. Review of the literature identifies delayed-onset seizure as a potential complication of quetiapine overdose. Unique to this case, delayed-onset seizures occurred in a patient with a relatively low dose of quetiapine and no obvious toxidrome, suggesting that this reaction may be an important consideration in the management of quetiapine overdose. The pharmacokinetics and pharmacodynamics of quetiapine may explain this unusual phenomenon.

## 1. Introduction

Quetiapine is an atypical antipsychotic that possesses low to moderate antagonist activity at multiple neurotransmitter receptors, including serotonergic 5-hydroxytryptamine (5-HT_2A_) receptors, dopaminergic (D_1_ and D_2_) receptors, histaminergic (H_1_) receptors, and adrenergic *α*_1_ and *α*_2_ receptors, and partial agonism at 5-HT_1A_ receptors [1]. Quetiapine has become widely used for a variety of indications because of its favorable side effect and safety profile, notably with decreased incidence of extrapyramidal symptoms compared to other antipsychotics. Patients with an acute overdose of quetiapine may demonstrate central nervous system depression, sinus tachycardia, prolonged QTc interval, hypotension, coma, and seizures [2–4]. We report a case of quetiapine overdose accompanied by a delayed-onset seizure, a rare and poorly understood complication.

## 2. Report of a Case

A 21-year-old woman was brought to the emergency department (ED) 22 hours after reporting an intentional ingestion of 1,400 mg of quetiapine. She had also consumed approximately 5 drink equivalents of alcohol. Within a few hours of her overdose, she became somnolent and slept for 19 hours until the next morning. When she awoke, the patient attended an outpatient psychiatry appointment where she disclosed her overdose and was subsequently brought to the ED. At the time of the ED evaluation, she denied any ongoing symptoms resulting from the overdose. Her daily medications were verified as 300 mg of bupropion XL and 50 mg of quetiapine, and her typical alcohol consumption was 2 drink equivalents per week. The patient had started quetiapine 4 days previously as part of a regimen for major depressive disorder; she did not have a known history of psychosis or manic symptoms and was previously quetiapine naïve. She denied a history of previous seizures.

In the ED, her vital signs were stable, with temperature at 36.9°C, blood pressure at 121/85, heart rate at 95 beats/minute, and respiratory rate of 16 breaths/minute. Her weight measured 80 kilograms. On examination, she was alert and oriented with no focal neurological deficits. A 12-lead ECG was unremarkable and her urine toxicology screen was negative for amphetamines, barbiturates, benzodiazepines, cannabinoids, cocaine, methadone, opiates, oxycodone, and ethanol. A basic metabolic panel and liver function panel were within normal limits. From the ED, she was deemed medically stable and admitted to the inpatient psychiatry ward.

In the psychiatry ward, six hours after presentation to the ED (thus approximately 28 hours after overdose), the patient experienced a generalized tonic-clonic seizure that was witnessed by a nurse, lasting 30 seconds and resolving spontaneously. Immediately following the seizure episode, the patient was somnolent with altered mental status. Initially, she was unable to state her name or current whereabouts and reported feeling confused and drowsy. She exhibited sinus tachycardia (130 beats/minute), temperature of 37.9°C, and QTc interval of 438 ms; computed tomography of the brain without contrast was normal. Prolactin level was noted to be mildly elevated at 28.3 ng/ml (reference range: 3–23.1 ng/ml). She was transferred to the medicine service for further monitoring, whereupon magnesium sulfate was administered and her postictal symptoms gradually resolved over several hours. An awake electroencephalography (EEG) was performed 23 hours postseizure and showed no evidence of epileptiform activity or other abnormalities. No additional seizure activity was noted during her stay. The remainder of her course was uncomplicated until her discharge 8 days later.

## 3. Discussion

Delayed or late-onset seizure is a rare complication of quetiapine overdose and has been reported in two previous case studies. Young et al. [5] reported a case of a 27-year-old woman who ingested 30 g of quetiapine and who had two generalized tonic-clonic seizures after 24 hours, each lasting less than 1 minute and treated with lorazepam and propofol. Yam et al. [6] described a 31-year-old man who ingested 4 g of quetiapine and was found unresponsive at home, presenting with a Glasgow Coma Scale score of 3. Twenty-seven hours following ingestion, he had a generalized tonic-clonic seizure lasting 45 seconds, treated with lorazepam and phenytoin. The unique aspect of this reported case includes the lower dose of quetiapine (1,400 mg), with fewer accompanying signs and symptoms of overdose.

The delayed-onset seizure is curious because its pathophysiology is difficult to explain. First, the presence of seizure is only a rare side effect of quetiapine overdose, even at relatively high doses. A chart review from the California Poison Control System database noted seizures in 22 of 945 of patients who had overdosed on quetiapine [3]. Second, the delayed occurrence of a seizure following overdose incident is peculiar in light of the pharmacokinetics of quetiapine. Other reports of seizure place the timing at 4–8 hours following quetiapine overdose [2, 7]. Plasma concentration of quetiapine typically peaks at 1–1.5 hours, and its elimination half-life is approximately 6 hours [8, 9]. Therefore, after 28 hours, quetiapine is present at less than 5% of its maximum plasma concentration. Delayed-onset seizures have also been rarely reported with overdose of other drugs with similar pharmacokinetic properties, for example, 13 hours after ingestion of 400 mg of citalopram [10], 9 hours after 6.75 g of bupropion extended release [11], up to 24 hours in a large series of bupropion XL overdoses [12], and 19 hours after ingestion of an unknown amount of amitriptyline [13]. However, these examples demonstrate a shorter interval than reported in our case and in other instances of quetiapine overdose.

This case provides new insight into the toxicity of quetiapine. Seizures, though a rare complication of atypical antipsychotic overdose, may be caused by blockade of D_2_ receptors [14], histamine receptors [15], *γ*-aminobutyric acid (GABA_A_) receptors, adrenergic receptors, and hypoxia [16]. Compared to other atypical antipsychotics (and in general, typical antipsychotics), quetiapine has a higher binding affinity to H_1_ receptors and a lower affinity for D_2_, 5-HT_1A_ and 5-HT_2A_ receptors [17, 18]. Given this spectrum of receptor binding affinities, we speculate that blockade of histamine receptors may mediate the delayed-onset seizures of quetiapine, although it is probable that this phenomenon arises out of interactions between receptor types. The action of quetiapine at these receptors may occur on longer time scales compared with plasma clearance. For example, the receptor occupancy half-life for D_2_ receptors was estimated at 10 hours, and the 5-HT_2_ effective occupancy half-life has been estimated at 27–64 hours [9, 19]. These properties may explain the unusual observation of delayed seizures in quetiapine overdose long after the peak plasma drug concentrations.

A limitation of this study is the generalizability of the patient's presentation to others with quetiapine overdose; therefore we considered other possible etiologies of seizure. The patient was young and healthy, without electrolyte abnormalities or structural defects on neuroimaging. Though she was regularly taking bupropion XL, a drug known to lower the seizure threshold, her dose was moderate (300 mg) and previously well-tolerated and therefore was unlikely to cause seizures. However, a contribution to quetiapine toxicity cannot be ruled out. Her alcohol use was also relatively low, and acute withdrawal was unlikely. Furthermore, the plasma concentration of quetiapine was not measured in this patient; therefore, it is unclear whether the patient's clearance or metabolism of quetiapine was abnormal. Indeed, the slight elevation in serum prolactin level at time of presentation may point to prolonged persistence of quetiapine. Future measurement of plasma quetiapine levels in patients presenting late may clarify the relationship between pharmacokinetics and late-onset seizures.

In this case, we report the occurrence of a delayed-onset seizure 28 hours following quetiapine overdose in the amount of 1,400 mg. Together with previous reports in the literature, this case illustrates the rare potential for delayed-onset seizures that may correlate with the unusual pharmacokinetics of quetiapine, providing new insight into pathophysiology. We suggest that providers who are evaluating or managing patients following quetiapine overdose should consider the possibility that the patient may be at risk for seizures for 24 hours (or longer), even in the absence of other symptoms of acute toxicity.
